# The Inhibition of the Highly Expressed Mir-221 and Mir-222 Impairs the Growth of Prostate Carcinoma Xenografts in Mice

**DOI:** 10.1371/journal.pone.0004029

**Published:** 2008-12-24

**Authors:** Neri Mercatelli, Valeria Coppola, Desirée Bonci, Francesca Miele, Arianna Costantini, Marco Guadagnoli, Elena Bonanno, Giovanni Muto, Giovanni Vanni Frajese, Ruggero De Maria, Luigi Giusto Spagnoli, Maria Giulia Farace, Silvia Anna Ciafrè

**Affiliations:** 1 Department of Experimental Medicine and Biochemical Sciences, University of Rome “Tor Vergata”, Rome, Italy; 2 Department of Hematology, Oncology and Molecular Medicine, Istituto Superiore Sanità, Rome, Italy; 3 Department of Biopathology, University of Rome “Tor Vergata”, Rome, Italy; 4 Department of Urology (LDU, GM), S. Giovanni Bosco Hospital, Turin, Italy; 5 Department of Internal Medicine, University of Rome “Tor Vergata”, Rome, Italy; 6 Mediterranean Institute of Oncology, Viagrande, Catania, Italy; Ordway Research Institute, United States of America

## Abstract

**Background:**

MiR-221 and miR-222 are two highly homologous microRNAs whose upregulation has been recently described in several types of human tumors, for some of which their oncogenic role was explained by the discovery of their target p27, a key cell cycle regulator. We previously showed this regulatory relationship in prostate carcinoma cell lines *in vitro*, underlying the role of miR-221/222 as inducers of proliferation and tumorigenicity.

**Methodology/Principal Findings:**

Here we describe a number of *in vivo* approaches confirming our previous data. The ectopic overexpression of miR-221 is able, per se, to confer a high growth advantage to LNCaP-derived tumors in SCID mice. Consistently, the anti-miR-221/222 antagomir treatment of established subcutaneous tumors derived from the highly aggressive PC3 cell line, naturally expressing high levels of miR-221/222, reduces tumor growth by increasing intratumoral p27 amount; this effect is long lasting, as it is detectable as long as 25 days after the treatment. Furthermore, we provide evidence in favour of a clinical relevance of the role of miR-221/222 in prostate carcinoma, by showing their general upregulation in patient-derived primary cell lines, where we find a significant inverse correlation with p27 expression.

**Conclusions/Significance:**

These findings suggest that modulating miR-221/222 levels may have a therapeutic potential in prostate carcinoma.

## Introduction

MicroRNAs are short (∼22 nt) RNA molecules whose relevance as regulators of gene expression has been shown in relatively recent times [1], during which, however, a huge amount of data have been collected demonstrating that they play extremely important roles in almost all aspects of biology, such as development and disease. They commonly act as negative regulators of the expression of protein coding genes, usually recognizing and binding to specific sites in the 3′UTRs of their mRNAs, and impairing their translation, or sometimes even inducing the degradation of the target mRNA (for an exhaustive review, see [2]. Their involvement in cancer onset and progression is to date well assessed [3], to such an extent that we can now classify microRNAs as “oncomiRs” (oncogenic microRNAs) or, conversely, tumor suppressor microRNAs [4]. Among oncomiRs, we and others previously found that miR-221 and miR-222 are involved in several different types of human neoplasms, such as glioblastoma [5]–[8], prostate carcinoma [9], non-small cell lung cancer [10], [11], hepatocellular cancer [12], [13], pancreatic cancer [14], and many others. The common observation was that this couple of microRNAs, or at least one of them, is significantly upregulated in tumors versus normal tissues, and often its expression marks the most aggressive forms of human solid tumors. The molecular basis of their “oncogenic” role was clarified for the first time by our group in the context of prostate carcinoma cells, through the discovery of their target mRNA, p27^kip1^, a negative regulator of cell cycle progression [9], and then this same finding was confirmed in most forms of cancers where the overexpression of miR-221/222 had been detected [7], [8], [11], [13], [15]. More recently, another cell cycle inhibitor, p57, has been described as a specific target of miR-221/222 [8], [13], once more contributing to the general rule that one microRNA can have pleiotropic effects by targeting more than one mRNA. In this way a single microRNA can control a whole biological (or pathological) pathway by “hitting” numerous of its keypoints. The reason accounting for the recognition of shared targets for both miR-221 and miR-222 is found in their “seed” sequences, short (∼7–8 nt) regions at their 5′ ends through which they bind their target sites in mRNA 3′UTRs: these “seeds” are identical in miR-221 and miR-222 and are also very well evolutionarily conserved, likely indicating the common involvement of these two microRNAs in the same pathways.

Prostate carcinoma represents a big challenge to the scientific and clinical community as it remains the most common malignancy in men of the Western world, where it is still the second leading cause of cancer death [16]. The study of the involvement of microRNAs in this tumor dates back to only three years ago [17], and the first evidence clearly linking a microRNA and its target to prostate carcinogenesis is even more recent [18]–[21]. We previously described that miR-221/222 expression is directly correlated with the aggressiveness of cell models of prostate carcinoma, and that the forced overexpression of miR-221 or miR-222 in the poorly aggressive prostate carcinoma LNCaP cell line is sufficient to accelerate their proliferation and *in vitro* tumorigenicity [9]. In the limited number of studies available to date involving patients tissues, the expression of some selected microRNAs has been proven useful as a biomarker for prostate carcinoma [22]–[24], but no results have been published about the possibility of *in vivo* modulating the expression of microRNAs which are deregulated in this tumor.

The aim of our work was to clarify if the overexpression of these microRNAs is able to enhance prostate carcinoma growth *in vivo*, as it is *in vitro*, in a mouse model of subcutaneously induced tumorigenesis, to provide a proof of the relevant role played by this microRNA also *in vivo*. On the other hand, we sought to investigate if it is possible to inhibit miR-221 and miR-222 expression in mouse models of established prostate carcinoma, in order to set up the premises for a future therapeutic approach. To achieve this goal, we treated pre-established tumors induced by the s.c. injection of PC3 cells into SCID mice, with anti-miR-221 and anti-miR-222 “antagomirs”, cholesterol-conjugated antisense molecules previously shown to own a good bioavailability and stability *in vivo* [25], [26]. Moreover, we wanted to validate the clinical relevance of miR-221/222 expression in prostate tumors, and thus we measured the expression of these two microRNAs in primary cells from 18 patients with stage II–III prostate cancer, and concurrently quantified p27 expression, to check if the inverse relationship linking miR-221/222 to p27 is reproducible and significant in clinical samples.

Our findings indicate that miR-221 and miR-222 are key modulators of prostate carcinoma also *in vivo*. In fact, their inhibition significantly slows down tumor growth in a mouse model, and they appear aberrantly expressed in patient samples compared to normal tissues.

## Results

### The overexpression of miR-221 is sufficient to strongly enhance growth of LNCaP xenografts

**Figure 1:**
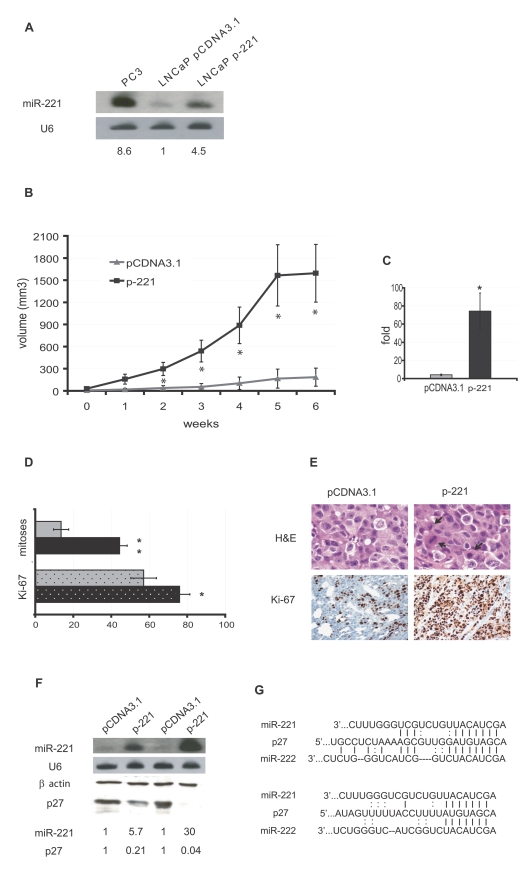
MiR-221 ectopic overexpression enhances the growth of LNCaP-derived tumors. A Northern blot analysis of LNCaP cells permanently transfected with p-221 or empty vector pCDNA3.1. The expression of miR-221 in the highly aggressive PC3 prostate carcinoma cell line is also shown, as a positive control. Hybridization to snRNA U6 is included as a loading control. Under each lane, a number indicates the relative miR-221 expression as compared to LNCaP cells transfected with the empty vector pCDNA3.1, where miR-221 endogenous expression is set as  = 1. B *In vivo* tumor growth in SCID mice. Average tumor volumes are represented (n = 6 for both experimental groups) starting from the first time point when tumor volumes were clearly measurable (t0) until the last measurement before sacrifice, performed 6 weeks later. C Average volume fold increase of the same tumors as in B at the moment of sacrifice (i.e. 6 weeks after the first measurement) as compared to values measured at time 0. Data are presented as the mean±SEM *, P<0.05, and are representative of 2 independent experiments. D–E Proliferation markers: mitotic index and Ki-67 expression. D Graph of the mitotic index and Ki-67 expression as percent of positive cells (10 fields, 2 sections for each tumor. *, P<0.01; **, P≪0.001). Grey bars: pCDNA3.1 transfected LNCaP cells; black bars: p-221 transfected LNCaP cells. E, left upper panel: LNCaP cells transfected with empty vector pCDNA3.1, haematoxylin eosin stained section (magnification 600×). Cells have epithelioid phenotype with low mitotic index. Right upper panel: tumor tissue from LNCaP cells transfected with p-221, haematoxylin-eosin stained section (magnification 600×). Cells have epithelioid phenotype with high mitotic index; arrows indicate mitotic pictures. Left lower panel: immunohistochemistry of the proliferation marker Ki-67 in tumor tissue from LNCaP ctrl cells; scattered cells with brown, granular nuclear staining considered to be positive for Ki-67 (magnification 200×). Right lower panel: immunohistochemistry of the proliferation marker Ki-67 in tumor tissue from LNCaP cells transfected with p-221: numerous cells with brown, granular nuclear staining positive for Ki-67 (magnification 200×). F Northern and Western blot analysis of RNA and proteins extracted from p-221 and control vector transduced tumors from two mice sacrificed at 6 weeks from the first measurement (as in B). The upper part of the panel (Northern blot) shows the persistent expression of miR-221 in p-221 transduced tumors, and the lower part (Western blot) shows the downregulation of p27 in miR-221 expressing tumors. U6 and β-actin are shown as loading controls for Northern and Western blot, respectively. The numerical values under each lane indicate the relative expression of miR-221 and of p27, where each p-221 transfected tumor is compared to its controlateral control (pCDNA3.1) tumor, whose miR-221 and p27 expression levels are set as  = 1. G p27 mRNA 3′UTR sites targeted by miR-221 and miR-222. The core annealing regions are located at nucleotides 201–208 and 274–281 of p27 3′UTR. Dotted vertical lines indicate G-U bonds.

We have recently shown that miR-221 and miR-222 are positive regulators of *in vitro* prostate carcinoma growth through the repression of p27 [9]. In our *in vitro* models, each microRNA can separately reduce p27 protein levels, and consequently is able to accelerate prostate carcinoma cell growth and colony formation [9]. In order to investigate if this role is relevant also in *in vivo* models of prostate carcinoma, we employed LNCaP cells permanently transfected with p-221 [9], expressing one of these microRNAs, miR-221 (**Fig. 1A**), to establish subcutaneous tumors in SCID mice, and measured tumor growth and significant features. As shown in **Fig. 1B**, a strongly significant increase in growth was conferred to tumors overexpressing miR-221, as compared to empty-vector transfected control tumors. As clearly depicted in the graph, the advantage in growth was very early achieved by miR-221 expressing tumors, producing volumes that were statistically much greater than control ones for the whole duration of the experiment (p<0.01 for all time points). In agreement with this observation, the average volume fold increase of miR-221 expressing tumors at the end of the experiment was extremely higher than that of control tumors (4.024±0.89 *vs* 74.432±19.79, p = 0.025) (**Fig. 1C**). Thus, the mere overexpression of miR-221 is able, *per se*, to highly enhance the growth of LNCaP xenografts.

### MiR-221 overexpressing tumors display significantly enhanced levels of proliferation markers and reduced p27 expression

To further characterize the proliferative status of miR-221 and control xenografts, we measured the mitotic index and the expression of Ki67 as markers of proliferation in the xenograft tumors. As shown in the representative micrographs of **Fig. 1E** (lower panels), there was a substantial increase in the percentage of Ki67 positively stained nuclei in tumors from the p-221 transfected cells, as compared with that in control tumors transfected with pCDNA3 (p221 = 76% *vs* CTRL 57% p<0.01; **Fig. 1D**). The mitotic index showed a similar trend with a three-fold increase in miR-221 expressing cells (p-221 = 44.4 *vs* CTRL 13.6, p<0.00003; **Fig. 1D,E**, upper panels).

In order to verify whether the effects observed in tumor growth and proliferation were due to a persistent expression of miR-221, we performed Northern blot analysis. As shown in **Fig. 1F**, the expression of miR-221 was still very strong, even though a long time had passed after the subcutaneous injection of LNCaP cells. This observation led us to check the p27 status in transfected tumors, in search of the inverse correlation expected on the basis of the *in vitro* validated negative regulation of p27 by miR-221 [9], exerted by miR-221 and miR-222 via the specific recognition of two target sequences in the p27 3′UTR (**Fig. 1G**). Western blot analysis performed on protein extracts from miR-221 expressing tumors showed a clear reduction of p27 levels, as compared to control samples (**Fig. 1F**). These data indicate that the persistent miR-221 overexpression reduces p27 expression and stimulates proliferation in LNCaP cell xenografts.

### The in vitro depletion of miR-221 and miR-222 renders PC3 cells less efficient in the establishment of in vivo xenografts

**Figure 2:**
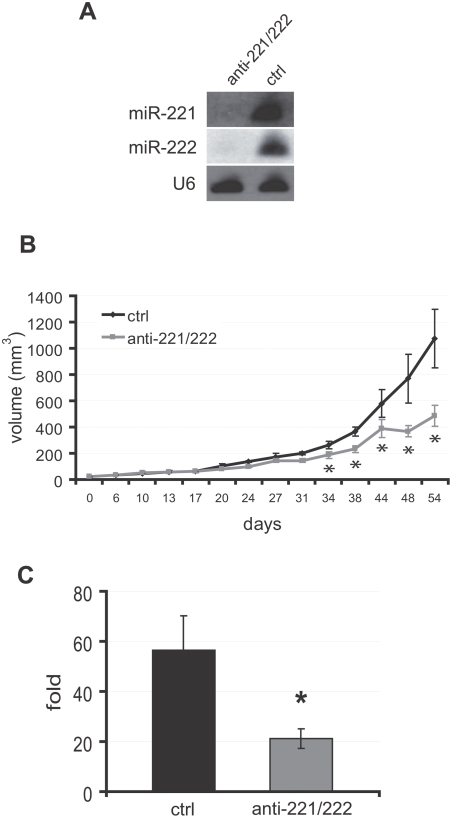
*In vitro* inhibition of miR-221 and miR-222 reduces tumor growth of PC3 derived tumors in SCID mice. A Northern blot analysis of total RNA extracted from PC3 cells transfected *in vitro* with anti-miR-221+anti-miR-222 LNA oligonucleotides (anti-221/222). The hybridisation to snRNA U6 was used as a loading control. B Tumor growth curves measured after the injection of PC3 cells transfected with either anti-miR-221 and anti-miR-222 LNA oligonucleotides (anti-221/222) or a control LNA oligo (ctrl). The tumor volumes were calculated as v = L×l^2^×0.5, where L is the longer diameter, and l the shorter one. C Average volume fold increase of tumors derived from PC3 cells transfected with anti-miR-221+anti-miR-222 LNA oligonucleotides (anti-221/222) or with a negative control LNA oligonucleotide (ctrl). Values represent the ratio between the volumes at the day of sacrifice and the volumes measured 54 days before, when all tumors were clearly detectable and measurable. N = 4 for ctrl tumors and n = 5 for anti-miR treated tumors. Data in B and C are presented as the means±SEM. *, P<0.05.

The data collected with LNCaP cells, physiologically expressing low levels of miR-221, represent a proof of principle that miR-221 is a powerful enhancer of prostate tumor growth. Thus, it can ideally represent a target for a treatment aimed at reducing tumor growth. As a first step to test this hypothesis, we pre-transfected PC3 cells, a high miR-221 and miR-222 expressing prostate carcinoma cell line [9], with LNA oligonucleotides targeting mir-221 and miR-222, in order to abolish their expression. As shown in **Fig. 2A**, LNA oligoes efficiently depleted miR-221 and mir-222 from PC3 cells, to such an extent that miR-221/222 expression was almost undetectable by Northern blot. Treated cells were then subcutaneously injected into SCID mice, and tumor growth was followed and compared to that generated by control LNA oligo-transfected PC3 cells. In **Fig. 2B** a negative modulation of growth is shown for miR-depleted tumors compared to control ones: the growth curves of control and pre-transfected tumors start diverging when control tumors begin their exponential growth, while the slope of pre-treated tumors is still very low, and remain statistically different (*, p<0.05) until the end of the experiment. When measuring the average volume fold increase of tumors at the sacrifice with respect to the first measurements performed, a statistically significant difference was detectable between treated and control tumors (**Fig. 2C**, 21.18±3.95 *vs* 56.41±13.75, p = 0.029).

### In vivo intratumoral knockdown of miR-221 and miR-222 upregulates p27 and reduces tumor growth of PC3 xenografts

**Figure 3:**
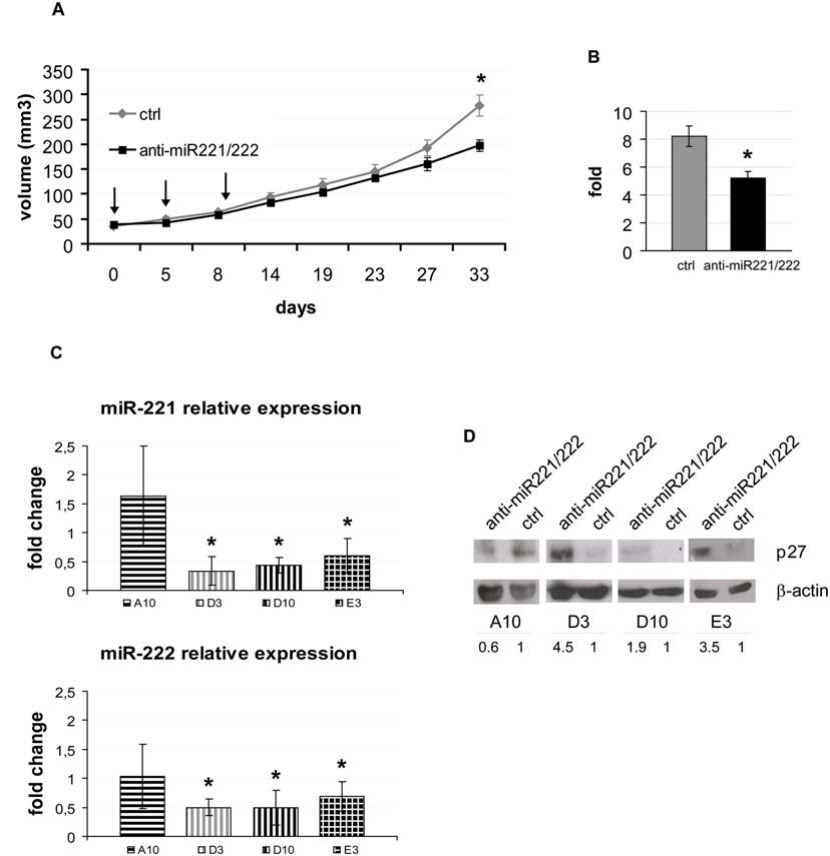
The intratumoral injection of anti-miR-221 and anti-miR-222 antagomirs into PC3-derived tumors reduces tumor growth and has long lasting effects on miR-221 and miR-222 endogenous expression. A Tumor growth curves depicting the average±SEM values of PC3 derived tumors injected either with a negative control antagomir (ctrl) or with a mixture of anti-miR-221 and anti-miR-222 antagomirs (anti-miR221/222). Each tumor was treated with three injections of 1 µg of each antagomir at days 0, 5, and 9 (arrows). Day 0 is the day of the first antagomir injection. Each mouse (n = 8) was bearing a negative control injected tumor on one flank, and an antagomir treated one on the controlateral flank. Data are presented as the means±SEM of two independent experiments. *, P = 0.009. B Average volume fold increase of the same tumors as in A. The data presented are the means±SEM, and represent the ratio between the volumes at the sacrifice (33 days from the first antagomir injection) and the volumes measured at the day of the first antagomir injection. *, P = 0.009. C Quantitative real-time PCR of miR-221 (upper panel) or miR-222 (lower panel) in tumors excised from four representative mice (A10, D3, D10, E3) at the day of sacrifice, 24 days after the last antagomir injection. The data are presented as the means±SD of three independent experiments, each performed in triplicate. *, P<0.05. The values presented are the miRNA expression fold changes, as compared to the expression detected in negative control tumors grown in each mouse, set as  = 1. D Representative p27 Western blot analysis on total proteins extracted from the same tumors as in c. For each mouse, the proteins from the negative control tumor and the antagomir-treated tumor are shown. β-actin immunoreactivity is shown as a loading control. Under each lane, the numerical values represent p27 relative amount, that was set  = 1 in each control tumor (ctrl), and the p27 expression in the respective controlateral anti-miR221/222 tumor was calculated.

Encouraged by the previous results, we took a further step toward the assessment of the feasibility of the direct *in vivo* anti-miR-221/222 treatment. PC3 cells were subcutaneously injected into both flanks of SCID mice in order to yield tumors that were then treated by direct intratumoral injection as soon as they became clearly palpable. For each mouse, the tumor on one flank was injected with a mixture of anti-miR-221 and anti-miR-222 antagomirs, while the controlateral tumor was injected with a control antagomir. The growth curves of treated *vs* untreated tumors are compared in **Fig. 3A**: as shown, the two curves slowly become divergent until they reach statistically different values at 33 days after the first antagomir injection (197.2±12.21 *vs* 276.82±21.28, p = 0.009). In accordance with this observation, the average volume fold increase of treated tumors at the end of the experiment with respect to the day of the first antagomir injection, set as day 0, was significantly reduced as compared to that of control tumors (5.2±0.5 vs 8.19±0.73, p = 0.009) (**Fig. 3B**). These numbers, in fact, represent the observation that 7 out of 8 experimental animals had a reduced volume fold increase at the day of sacrifice for the treated tumor with respect to the controlateral control tumor. In this experimental setting, where each mouse bears both the treated tumor and the control one, thus avoiding the common high inter-animal variations linked to the assignment of different treatments to different groups of animals, this result obtained for the volume fold increase appears particularly encouraging.

We then sought to determine if the observed effects on tumor growth were still accompanied, at the day of sacrifice, by a consistent reduction of miR-221/222. To answer this question, we performed Q-RT-PCR on total RNA extracted from excised tumors, and verified an effective and persistent reduction of miR-221 and miR-222 in treated tumors *vs* control ones (**Fig. 3C**).

We also checked if antagomir-mediated suppression of miR-221/222 resulted in a correspondent increase of p27 levels, as compared to untreated tumors; **Fig. 3D** shows a representative image of Western blot analyses of total protein extracts from the same tumors already tested for miR-221/222 expression: in all cases assayed, a high p27 expression was measured where miR-221 and miR-222 were kept low by antagomir action, whereas the lack of inhibition of the two microRNAs matched with a low level of p27 expression.

Altogether, these results indicate that intratumoral injection of antagomirs targeting miR-221 and miR-222 can effectively keep low the concentration of these two microRNAs for as long as 24 days (i.e. time elapsed from third and last antagomir injection to animal sacrifice), concomitantly increasing p27 amount and ultimately reducing the growth of PC3 xenografts.

### MiR-221 and miR-222 are highly expressed in human prostate carcinoma primary samples and their expression is inversely correlated to that of p27

**Figure 4:**
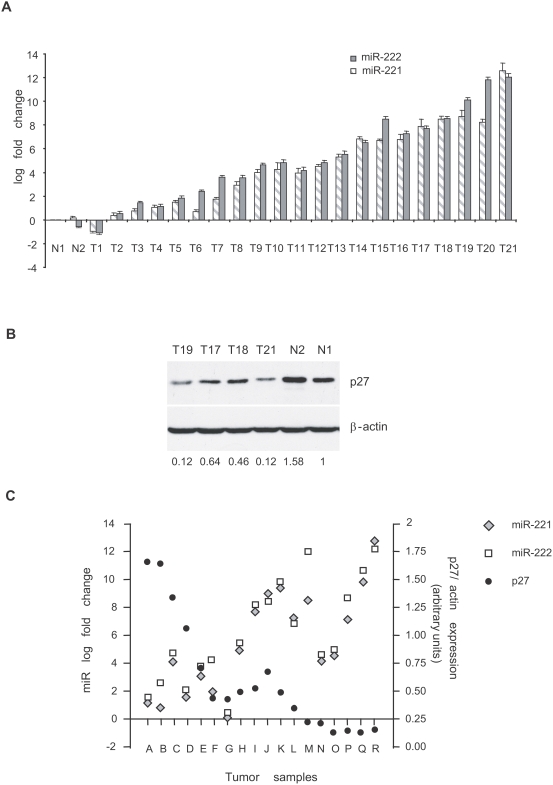
MiR-221 is strongly expressed in prostate carcinoma-derived primary cells and its expression inversely correlates with that of p27. A MiR-221 and miR-222 expression measured by quantitative real-time PCR in primary cell lines from prostate carcinomas (T samples) or normal prostate (N samples). The graph shows the log fold change of miR-221 and miR-222 expression as compared to the value obtained for non-tumoral control sample N1. B Representative Western blot analysis showing p27 expression in 4 tumor- and 2 non-tumor-derived primary cell lines. β-actin immunoreactivity is shown as a loading control. Under each lane, the numerical values represent p27 relative amount, that was set  = 1 in the normal control sample N1. C Spearman correlation analysis performed between miR-221, miR-222, and p27 levels in 18 primary cell lines derived from prostate carcinoma tissues. P<0.05.

To assess the significance of our results in human tumor samples, we analyzed miR-221 and miR-222 expression in 21 patients with stage II–III prostate cancer. Freshly-isolated surgical tumor specimens were collected and cultivated in a medium that allowed the propagation of prostate primary cells (see methods). Non tumor samples were used as control reference. Real-time PCR showed a consistent upregulation of both miR-222 and miR-221 in about 80% of the tumor samples analyzed with respect to normal counterparts, even if no correlation was observed with Gleason and stage (**Fig. 4A** and Table 1). The expression of the two microRNAs appeared always comparable, with no indication of a specific regulation of a single microRNA. We then analyzed the correlation between miR-221/222 and p27 in these patient samples by Western blot (**Fig. 4B**), and found that tumor samples characterized by high miR-221/222 had a significantly low amount of p27. When the values of p27 protein expression were plotted against miR-221 and miR-222 expression, an inverse correlation was evident (**Fig. 4C**, Spearman: p = 0.0164 for miR-221 and p = 0.0057 for miR-222). These results provide a strong indication that the previously identified regulatory relationship inversely linking miR-221/222 to p27 is true and relevant in human primary prostate carcinoma samples.

**Table 1:** Clinical aspects of prostate cancer cases used in this study.

Case^a^	Age	PSA^b^		Gleason grade^c^	pTNM^d^
		5–10 ng	>10 ng		
T1	74	5.7		Gleason 6	T3N0Mx
T2	58		94	Gleason 9	T3N0Mx
T3	69		44	Gleason 7	T3N0Mx
T4	65	7.2		Gleason 6	T2N0Mx
T5	71		14.45	Gleason 7	T3N0Mx
T6	54	3.9		Gleason 6	T3N0Mx
T7	59	9.5		Gleason 6	T3N0Mx
T8	63		15.16	Gleason 7	T3N0Mx
T9	61	6		Gleason 6	T2N0Mx
T10	58	9		Gleason 7	T2N0Mx
T11	67	7.1		Gleason 6	T2N0Mx
T12	60	10.2		Gleason 6	T2N0Mx
T13	57	7.3		Gleason 6	T2N0Mx
T14	62	8.6		Gleason 7	T2N0Mx
T15	63		5.9	Gleason 6	T2N0Mx
T16	58		12.15	Gleason 6	T2N0Mx
T17	67		12.18	Gleason 6	T2N0Mx
T18	69		12	Gleason 8	T2N0Mx
T19	57	6.5		Gleason 6	T2N0Mx
T20	69	6.13		Gleason 6	T2N0Mx
T21	70		10.13	Gleason 8	T3N0Mx

a Case: patients treated with radical prostatectomy.

b PSA: Prostate Specific Antigen.

c Gleason grade: Gleason's score.

d TNM: Tumor, Nodes, Metastasis.

pT: pT category, N: lymphnodes, N0: not involved; M: metastasis, Mx: not reliable.

## Discussion

Prostate carcinoma represents a field of great interest for the scientific and clinical community as it is, in its most aggressive forms, untreatable and the second leading cause of cancer death in men from the Western world. Effective treatments are still missing for the most aggressive forms of this tumor, and, also, there is still a great need for precise molecular markers of prostate carcinoma. In the recent years, the data collected about tumor-specific microRNA expression have shed a very promising light on the possibility of classifying some tumors on the basis of specific microRNA expression, raising new hopes about the use of microRNAs as diagnostic and prognostic tools [27]. In our work we aimed to demonstrate that the overexpression of miR-221 and miR-222, a couple of microRNAs that we had previously shown to be strongly upregulated *in vitro* in aggressive prostate carcinoma cell lines, is relevant to prostate carcinoma cell growth *in vivo*, both in mouse models and in human tumor samples. To achieve this, we have overexpressed miR-221 in the poorly aggressive human prostate carcinoma cell line LNCaP, and observed the growth of tumor xenografts derived from those cells in SCID mice. Our results are in full agreement with the pro-proliferative action of miR-221: tumors grew faster and larger, their mitotic and proliferative (Ki-67) indexes were strongly enhanced, and the long-lasting overexpression of miR-221 reduced the tumor expression of p27, again confirming our *in vitro* data.

However, of course, the great interest of this observation lies in its reverse implications: that inhibiting miR-221 and miR-222 in prostate carcinoma may be a way to reduce its growth potential. Indeed, we have demonstrated this *via* two different approaches whose results anyhow converge towards the same conclusion. We have pre-transfected cells from the highly aggressive PC3 cell line with LNA antisense oligonucleotides targeting miR-221 and miR-222, and subsequently followed the growth of tumor xenografts obtained through the injection of pre-transfected cells into SCID mice. On the other hand, we have injected anti-miR-221 and anti-miR-222 antagomirs into pre-established PC3 xenografts. Both approaches clearly aimed at reducing miR-221 and miR-222 in the tumors but, while the first one theoretically conferred a delay to pretransfected cells that received the LNA oligoes, before they settled in the host environment and started assembling a true tumor, the second one more closely mimicked a “treatment”, as it was performed in already grown tumors, where cells had already formed their network of contacts within the host body. For these reasons we believe that our positive data in this latter settlement are the most significant and interesting, as they show that miR-221/222 inhibition can reduce the growth of pre-established prostate carcinoma xenografts. Once more, we show that treated tumors growing smaller than controls maintain reduced levels of miR-221 and miR-222 for the whole duration of the experiments, and that this produces a permanent upregulation of p27, otherwise low in control tumors. Thus, our prostate carcinoma xenograft data demonstrate, as a whole, that miR-221 (and most likely miR-222, even if here we are not providing a direct evidence for this) is sufficient to strongly enhance prostate carcinoma growth and, consequently, that the inhibition of miR-221 and miR-222 is necessary, and in fact effective, to reduce the *in vivo* growth of this tumor. We think that our data involving antagomirs, beside being promising *per se*, represent one of the first attempts to use these molecules locally in a tumor. In fact, while most of the papers published to date provide data about the systemic use of use of antagomirs [26], [28], only two recent papers have described the intra-tumoral treatment with antagomirs: Felicetti and colleagues [29] employed antagomir doses similar to the ones we have used here, to treat melanoma xenografts, obtaining inhibitory results comparable to ours, even if the growth of the tumors was not followed for as long as we did in our present work. In a different work, Fontana and colleagues [30] injected anti-miR-17-5p antagomirs into neuroblastoma xenografts, indeed obtaining a total regression of 30% of tumors. It must be noticed, though, that the antagomir dosage employed in that work is several orders of magnitudes higher than the one we have used here. Moreover, the molecular analyses demonstrating the effectiveness of antagomir treatment were performed at very short times after antagomir injection, whereas we were able to prove an effective downregulation of miR-221/222, and a consequent upregulation of p27, for longer than three weeks.

Finally, we think that the relevance of our data, collected in mouse models of prostate carcinoma, is supported by the last results we show in this study, about a significant inverse correlation between miR-221/222 and p27 expression in primary cell lines derived from tumor samples of prostate carcinoma. Reduced p27 expression was associated with the most aggressive forms of prostate cancer and poor survival [31], [32], to such an extent that p27 was proposed as a biomarker for this tumor [32]. However, we still lack a full comprehension of the regulatory mechanisms perturbing p27 expression during prostate carcinoma onset and progression. We believe that our work now indicates that miR-221/222 upregulation may be one of the possible mechanisms responsible for p27 downregulation in this tumor. Further studies are certainly needed to more deeply dissect miR-221/222 role, also taking into account that it is very likely that their oncogenic action is not limited to the inhibition of p27, as recently demonstrated in other tumors [8], [13]. However, our results represent the first step toward a possible use of miR-221/222 as molecular markers for prostate carcinoma, and set the base for the future employment of anti-miR-221/222 antagomirs for the inhibition of prostate carcinoma growth.

## Materials and Methods

### Cell lines and transfections

LNCaP and PC3 cell lines were maintained in RPMI-1640 medium supplemented with 10% heat-inactivated fetal bovine serum, 20 mM L-Glutamine, 100 U/ml of penicillin G sodium, 100 µg/ml of streptomycin sulphate in a humidified atmosphere containing 5% CO_2_ at 37°C.

Transfections were performed by Lipofectamine 2000 reagent (Invitrogen, Italy) using 8 µg of plasmid DNA in Opti-MEM I (Invitrogen, Italy), as recommended by the manufacturer.

### In vivo studies

For studies involving LNCaP cells, 3×10^6^ exponentially growing, empty vector-transduced or miR-221-transduced LNCaP cells were resuspended in a solution of 50% Matrigel in PBS, and s.c. injected into the left and the right flank respectively of 5 wk old male CB.17 SCID mice (Harlan Italy S.r.l.).

For the experiments with *in vitro* transfected PC3 cells, LNA oligonucleotides against miR-221 and miR-222, and a negative control oligonucleotide were obtained from Ambion Inc. (Celbio, Italy). Knockdown oligos were transfected by Lipofectamine 2000 (Invitrogen, Italy) into PC3 cells at a final concentration of 40 nM each. A FAM-labeled negative control LNA oligonucleotide (Ambion, # AM 17012) was transfected in the same conditions as those used for the unlabeled LNA molecules, and used for measuring transfection efficiency by fluorescence microscopy at 48 hours after transfection. Transfection efficiency was estimated as 80–90%. After 48 hr from transfection, the cells were collected and miR-221 expression was analyzed by Northern blotting to verify the effective miRNA knockdown. At the same time point, 1.5×10^6^ cells were resuspended in PBS and s.c. injected into each flank of 5 wk old male CB.17 SCID mice (Harlan Italy S.r.l.). Each animal received control cells on one flank and anti-miR-221+anti-miR-222 pre-treated cells on the other.

For *in vivo* antagomir treatments, 5 wk old male CB.17 SCID mice (Harlan Italy S.r.l.) were s.c. injected in both flanks with 1.5×10^6^ wild type PC3 cells. After approximately one week, when the tumors reached an average volume of ∼50 mm^3^, the tumors were directly injected with a cocktail of antagomirs (Dharmacon, CelBio, Italy) targeting miR-221 and miR-222 on one flank, or with a control antagomir on the other. 40 µl of PBS containing 1 µg of each anti-miR-221 and anti-miR-222 antagomir, or control antagomir, were injected intratumorally at day 0, 5 and 9, for a total of three injections per tumor. Antagomir sequences were 5′-g_s_a_s_aacccagcagacaaugu_s_a_s_g_s_c_s_u-Chol 3′ (anti-miR-221), 5′-g_s_a_s_gacccaguagccagaugua_s_g_s_u_s_c_s_u-Chol 3′ (anti-miR-222). Lower case letters represent *2*′-*O*-Methyl-modified oligonucleotides, subscript ‘s’ represents a phosphorothioate linkage, and “Chol” represents 3′-linked cholesterol.

At the end of each study, animals were sacrificed and tumors were collected and divided into one part that was stored in RNA*later* (Ambion Inc., Celbio, Italy) following manufacturer's instructions for the following RNA extraction, and in another part, fixed in formalin for immunohistochemistry.

Animals were housed at the University animal house according to institutional guidelines, and all experiments were approved by the Institutional review Board. Tumor growth was monitored by caliper measurement once or twice a week for at least 5 weeks. Tumor volume was calculated as follows: V = L×l^2^×0.5, where L and l represent the larger and the smaller tumor diameter respectively.

### RNA extraction and Northern blot analysis

Total RNA was extracted from PC3 and LNCaP cells, and from excised tumors, by using Trizol reagent (Invitrogen, Italy) according to the manufacturer's instructions. For Northern blot analysis of miRNAs, 15 µg of total RNA were separated on 10% denaturing polyacrylamide gels and electro-transferred to Immobilon-Ny+ membrane (Millipore Corporation). The specific probes, end-labeled with T4 polynucleotide kinase in the presence of γ-^32^P-ATP, were: miR-221, 5′-gaaacccagcagacaatgtagc-3′; miR-222, 5′-gagacccagtagccagat-3′; U6, 5′-cacgaatttgcgtgtcatccttgcgcaggggcc-3′. Bands were quantified with ImageJ 1.34 s or OptiQuant 3.1 Packard Instrument software.

### Immunoblot Analysis

20–40 µg of whole cell protein extracts (lysis buffer: 20 mM Tris/HCl pH 7.2, 200 mM NaCl, 1% NP40 with Protease and Phosphatase Inhibitor Cocktails I and II (Sigma-Aldrich, Italy) were separated on 12% SDS-PAGE gels and transferred to nitrocellulose membrane. The levels of p27 expression were evaluated out by using the monoclonal anti-p27 antibody (610241; B&D Bioscience). As a loading control, β-actin expression levels were measured by rabbit polyclonal anti-actin antibody (A2066 Sigma, Italy) or anti-β-actin monoclonal antibody (Oncogene Research Products, San Diego, CA). The secondary horseradish peroxidase conjugated antibody (AP132P or AP160P; Chemicon) was detected using ECL Plus Western blotting detection reagents (Amersham Biosciences, Italy). Bands were quantified with ImageJ 1.34 s or OptiQuant 3.1 Packard Instrument software.

### Histology and Immunohistochemistry

The tumors were excised, fixed in a 10% paraformaldehyde solution, embedded in paraffin, and cut into 5 mm-thick slices for staining. A set of slides was stained with haematoxylin eosin for the morphological study and for the count of mitosis. To evaluate the mitotic index (number of mitotic pictures in ten high-power fields) ten fields at ×600 magnification were randomly selected and the number of mitosis counted. Another set of slides was stained with rabbit monoclonal antibody to Ki67 (Ventana Medical System Inc., Tucson, AZ, USA) to observe the proliferating cells, and the slides were viewed on a Nikon Eclipse E600 microscope (Nikon Corporation,Tokyo, Japan). The Ki-67 labeling index was determined by counting 1000 tumor cells at ×60 magnification. Ten high power fields, each containing 150–200 cells, were randomly selected for counting on each slide at a ×600 magnification. Brown, granular nuclear staining was considered to be positive for Ki-67. Labeling indices were calculated as the percentages of tumor cells with positive nuclear staining. Immunostaining was performed blindly and scored on prostate tumor tissue sections from each mouse (n = 10 per group; two sections from each tumor) by an independent pathologist. The number of positive cells over the total number of cells were counted manually by two independent operators. Both the Ki67-stained and unstained cells were counted, and the number of Ki67-positive cells per total number of cells determined the Ki67 score. For statistical evaluation, the Ki67 scores and the mitotic index were averaged over ten fields in each tumor and averaged again over all tumors in each group.

### Primary prostate cells

Tissues were obtained from radical prostatectomy at the Department of Urology, S. Giovanni Bosco Hospital of Turin, Italy, where this study was approved by the Institutional Review Board. All samples were collected with written, informed consent of the patients. For a detailed description of patients' samples, see Table 1. The tumoral or non-tumoral nature of each sample was determined by histopathological examination. Freshly-isolated surgical tumor specimens were collected and treated with collagenase for enzymatic dissociation. The homogenate suspensions were maintained in culture in collagen-coated plates with a specific medium that allowed the propagation of primary prostate cells (BRFF-HPC1 medium, AthenaES, Baltimore, MD). In these conditions, cells grew in a monolayer assuming a round-shaped aspect visible under the microscope. To determine the number of luminal cells and contaminating fibroblasts, cells were stained for cytokeratin 18 (Clone 5D3 by NovoCastra, used 1∶10) and Thy-1 (Clone 5E10 by Becton Dickinson, used 1∶50), respectively. The percentage of tumor cells was evaluated with anti-AMACR (1∶50, Sanova Pharma, Vienna, Austria), while normal basal cells were detected with anti-p63 (1∶50, BioGenex). Only cultures with >85% enrichment of prostatic epithelial cells were used for further experiments.

### MiR-221/222 quantitative real time PCR

Total RNA was extracted using TRIzol method. Fifty nanograms of RNA were reverse transcribed with M-MLV reverse transcriptase (Invitrogen, Italy) and cDNA was diluted 1∶10 in the PCR reactions. Housekeeping gene reverse transcription was performed using random primers, while miR specific looped-primers were used for miR-221 and miR-222 reactions. TaqMan microRNA assays (Applied Biosystems, Italy) for miR-221 and miR-222 were used for PCR amplification. Normalization was performed using rRNA S18 as a reference (S18 TaqMan assay on demand, Applied Biosystems, Italy). Calibration was performed using cDNA samples from normal prostate primary cells. Values are expressed in terms of 2-ΔΔCT where ΔΔCT = ΔCTsample−ΔCTcalibrator. ΔCT was the difference in threshold cycles between the miR and S18 amplicons, and CT was a parameter given by ABI PRISM 7700 Sequence Detector software by negative correlation with an internal reference dye (ROX).

### Statistical analysis

Results of quantitative analysis are presented as means±standard error (S.E.) or ±standard deviation (SD) as specified in the figure legends. Student's paired *t*-test was used to evaluate individual differences between means. Spearman correlation analysis was performed between miR-221/222 and p27 levels. P<0.05 was considered significant in all tests.
